# Inteins—mechanism of protein splicing, emerging regulatory roles, and applications in protein engineering

**DOI:** 10.3389/fmicb.2023.1305848

**Published:** 2023-11-08

**Authors:** David W. Wood, Marlene Belfort, Christopher W. Lennon

**Affiliations:** ^1^William G. Lowrie Department of Chemical and Biomolecular Engineering, The Ohio State University, Columbus, OH, United States; ^2^Department of Biological Sciences and RNA Institute, University at Albany, Albany, NY, United States; ^3^Department of Biological Sciences, Murray State University, Murray, KY, United States

**Keywords:** conditional protein splicing, expressed protein ligation, homing endonuclease, intein, mobile genetic element, posttranslational protein regulation, protein semi-synthesis, protein trans-splicing

## Abstract

Protein splicing is a posttranslational process in which an intein segment excises itself from two flanking peptides, referred to as exteins. In the native context, protein splicing results in two separate protein products coupled to the activation of the intein-containing host protein. Inteins are generally described as either full-length inteins, mini-inteins or split inteins, which are differentiated by their genetic structure and features. Inteins can also be divided into three classes based on their splicing mechanisms, which differ in the location of conserved residues that mediate the splicing pathway. Although inteins were once thought to be selfish genetic elements, recent evidence suggests that inteins may confer a genetic advantage to their host cells through posttranslational regulation of their host proteins. Finally, the ability of modified inteins to splice and cleave their fused exteins has enabled many new applications in protein science and synthetic biology. In this review, we briefly cover the mechanisms of protein splicing, evidence for some inteins as environmental sensors, and intein-based applications in protein engineering.

## 1. Introduction

**Figure 1:**
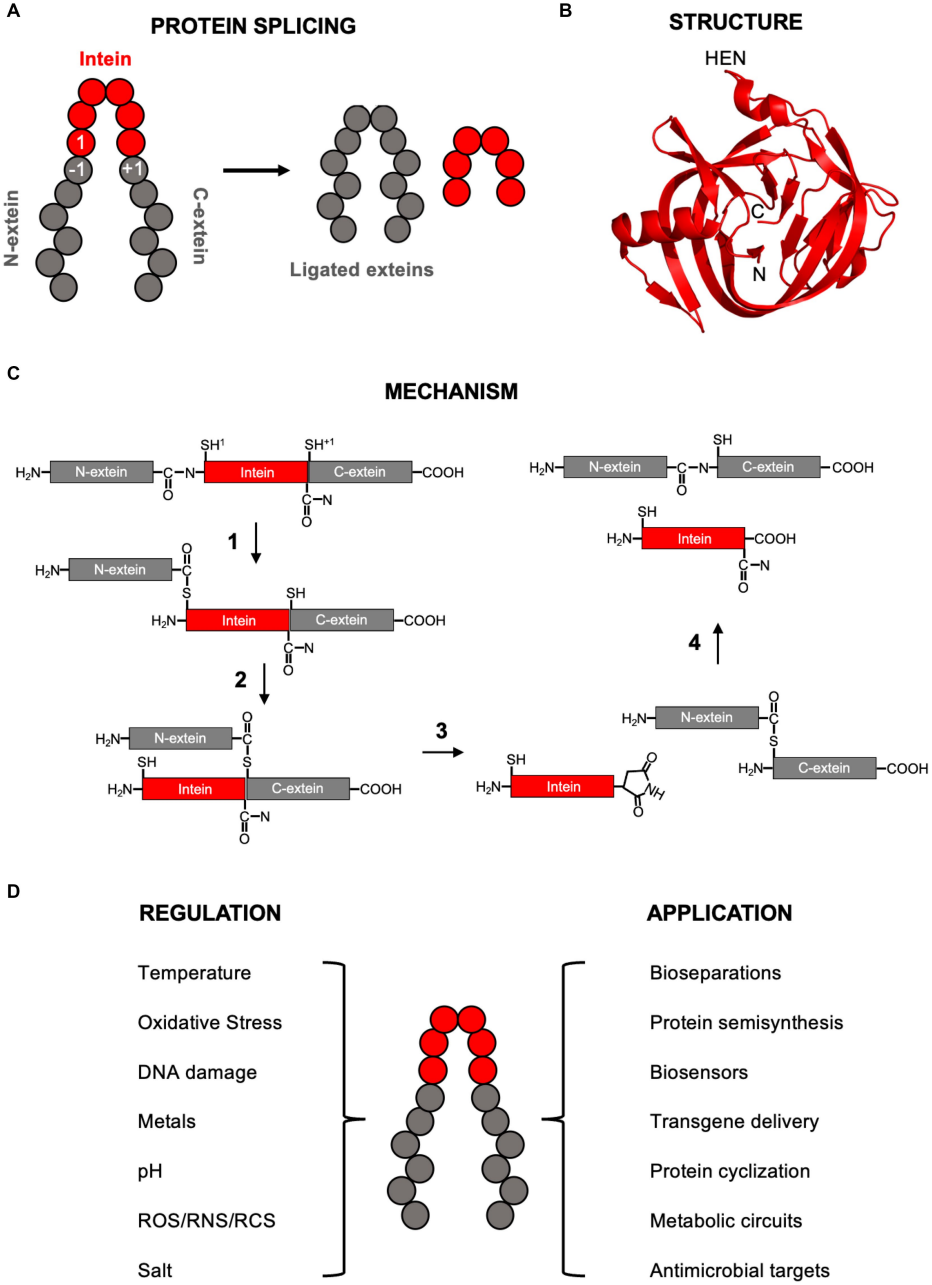
Intein background. **(A)** Simplified diagram of protein splicing with the exteins, intein, and −1, 1, and +1 residues labeled. **(B)** Structure of the RadA mini-intein from *Pyrococcus horikoshii* (PDB4E2T from Oeemig et al., 2012). The N-terminus, C-terminus, and site of homing endonuclease domain (HEN) insertion within full-length inteins are indicated. Image generated using Pymol. **(C)** Class 1 protein splicing mechanism. Each step explained in detailed in the main text. Briefly, in step 1 the cysteine at the 1 position makes a nucleophile attack on the preceding amide bond, resulting in a linear thioester. In step 2, the cysteine at the +1 position makes a second nucleophilic attack on the linear thioester formed in step 1, resulting in branched intermediate formation. In step 3, the terminal intein asparagine cyclizes, releasing the intein. In step 4, the thioester connecting the N- and C-exteins rearranges to an amide bond and the cyclized intein asparagine is hydrolyzed. In this scheme, the exteins are black, the intein is gray, and the 1 and +1 nucleophiles are cysteines (indicated by superscript). **(D)** Some environmental conditions known to regulate protein splicing (left) and applications of inteins (right). ROS, RNS, and RCS are reactive oxygen species, reactive nitrogen species, and reactive cholorine species, respectfully. In all panels, inteins are red and exteins in gray.

Inteins (**in**tervening pro**teins**) are translated within host proteins and removed in a self-catalyzed protein splicing reaction that simultaneously ligates the flanking sequences, known as N- and C-exteins, with a native peptide bond (Figure 1A). Since their discovery over three decades ago, inteins have proven exceptionally useful in protein engineering, have generated interest as novel antimicrobial targets, and have recently emerged as novel posttranslational regulatory elements (Volkmann and Mootz, 2013; Shah and Muir, 2014; Belfort, 2017; Lennon and Belfort, 2017; Prabhala et al., 2022).

The discovery of protein splicing arose from observations of large in-frame insertions in otherwise known host proteins during sequencing studies (Hirata et al., 1990; Kane et al., 1990). In each case, DNA sequence alignments could easily identify the flanking segments of the host protein, but their genes contained insertions that looked more like homing endonucleases. In most cases, the interrupted host protein was not observed (due to rapid splicing as soon as it was translated), which led to initial suspicions that the insertions were actually a new class of self-splicing introns. Curiously however, the insertions formed a continuous reading frame with the host protein segments, and single base deletions within the insertions led to a complete loss of the mature host protein activity. Ultimately it was proven the insertions, now known as inteins (Perler et al., 1994), are translated with the host protein and splice posttranslationally, leading to new research in their activity, biological significance, and applications.

Inteins have an overall fold that resembles a horseshoe, primarily made of β-sheets and loops, with the amino and carboxy ends of the intein brought in close proximity to one another to assist in ligation of flanking exteins (Figure 1B; Eryilmaz et al., 2014). Inteins are members of the Hint (Hedgehog-Intein) domain superfamily and bear structural similarity to the hedgehog C-terminal autoprocessing domain important during embryonic cell differentiation in animals (Kandel and Wang, 2022).

Most inteins are full-length, meaning there is a homing endonuclease domain (HEN) present between conserved regions known as splicing blocks (A, B, F, and G). This HEN domain (blocks C, D, E, and H) promotes intein spread through horizontal transfer into intein-minus alleles, making many inteins mobile genetic elements. The HINT and HEN domains are able to function independently of one another and the HEN domain can be deleted with retention of splicing activity, although the presence of the HEN domain can influence protein splicing (Derbyshire et al., 1997; Robinzon et al., 2020). Of full-length inteins, it is unclear what fraction house active HEN domains, as some have lost the ability cleave DNA at the expected site (Kelley et al., 2016).

In the case of mini-inteins, the HEN has been lost completely, leaving only the HINT domain. Remarkably, some mini-inteins, and therefore intein-housing genes, are split on host chromosomes and expressed as separate polypeptides. As a consequence, these split halves must meet within the cell to allow for *trans* splicing. While split inteins are rare in nature, inteins seem generally amenable to the introduction of artificial split sites (Aranko et al., 2014).

## 2. Mechanism of protein splicing

Several excellent reviews are available that provide an in-depth description of the mechanism of protein splicing (e.g., Volkmann and Mootz, 2013; Eryilmaz et al., 2014; Mills et al., 2014, and references within). This review will briefly cover the overall canonical class 1 mechanism (Figure 1C), important residues that facilitate protein splicing, and related but alternative strategies. Three classes of inteins have been described, each with similarities and difference in their mechanisms of protein splicing (Mills et al., 2014). In addition to being the most abundant, class 1 inteins are by far the best characterized, with an overall understanding of the steps for over two decades (Xu and Perler, 1996). When detailing the mechanism of protein splicing, by convention numbering of intein and extein residues is intein-centric, with the first amino acid of the intein is known as the 1 position, the last residue of the N-extein positions as the −1 position, and the first residue of the C-extein as +1 position (Figure 1C).

### 2.1. Canonical splicing by class 1 inteins

To begin the class 1 protein splicing reaction, the first residue of the intein, either a cysteine or serine, initiates a nucleophilic attack on the carbonyl carbon of the amide bond between the final residue of the N-extein (−1 position) and first residue of the intein (1 position) (Figure 1C). This results in an N-to-S or N-to-O acyl shift from the amide bond to either a thioester or ester, depending on if cysteine or serine, respectively, is in the 1 position. Highly conserved residues assist in the step 1 nucleophilic attack to form the linear (thio)ester, including threonine and histidine residues in the “TXXH motif” of Block B, and an aspartate in Block F. Conformational strain on the amide bond between the −1 and 1 positions that promotes the first step of splicing was observed in a redox trapped precursor (Callahan et al., 2011), which is mediated through hydrogen bonding by the Block B threonine (Eryilmaz et al., 2014; Mills et al., 2014). The Block B histidine, the most highly conserved intein residue, also contributes to the N-to-O/S acyl shift during linear (thio)ester formation and is essential for this step in many, but not all, inteins (Friedel et al., 2019). Mutation of this histidine can be compensated by certain residues in the −1 position (Friedel et al., 2019). Finally, the Block F aspartate stabilizes the deprotonated cysteine or serine side chain in position 1 to promote the acyl shift (Eryilmaz et al., 2014; Mills et al., 2014).

In step 2, the first residue of the C-extein (+1 position), a cysteine, serine, or threonine, performs a second nucleophilic attack on the linear (thio)ester bond formed in step 1 (Figure 1C). Deprotonation of the +1 residue side chain is required for this nucleophilic attack on the linear (thio)ester and the Block F aspartate forms hydrogen bond important to the trans-(thio)ester formation (Eryilmaz et al., 2014; Mills et al., 2014). This step 2 transesterfication results in formation of a branched intermediate, whereby the N-extein is bound by a (thio)ester to the C-extein 1 position side chain, and the intein is linked to the C-extein via an amide bond (Figure 1C).

In step 3, the last residue of the intein, an asparagine in Block G, cyclizes to release the intein from the branched intermediate (Figure 1C). This is facilitated by a nucleophilic attack on the carbonyl carbon of the amide bond between the intein and C-extein by the nitrogen of the asparagine side chain resulting in an aminosuccinimide (Eryilmaz et al., 2014; Mills et al., 2014). This step is also assisted by two histidine residues, one in Block G next to the terminal asparagine, and one in Block F. Not all inteins strictly require an asparagine as the terminal residue, using either glutamine or aspartic acid instead (Eryilmaz et al., 2014; Mills et al., 2014).

At this point, the N- and C-exteins are separated by a (thio)ester. In step 4, this (thio)ester undergoes an acyl rearrangement to form an amide bond, resulting in mature ligated exteins (Figure 1C). Additionally in step 4, the aminosuccinimide on the C-terminus of the intein is hydrolyzed to an asparagine (Eryilmaz et al., 2014; Mills et al., 2014) (Figure 1C).

### 2.2. Splicing in class 2 and class 3 inteins

The mechanism of class 2 protein splicing proceeds in three steps rather than the canonical four (Mills et al., 2014). Class 2 inteins lack the position 1 nucleophilic residue. Instead, a cysteine in the +1 position initiates the splicing reaction through attack on the amide bond at the junction between the N-extein and intein, leading to branched intermediate formation in a single step. Following this, the remaining two steps occur in the same manner as steps 3 and 4 for class 1 inteins.

Rather than the initiating nucleophilic residue at position 1, class 3 inteins utilize an internal Block F cysteine residue to begin the splicing reaction. In a single step, this Block F cysteine forms branched thioester intermediate. Next, the N-extein is transferred to the +1 position residue side chain, forming a second branched intermediate resembling that formed by class 1 and 2 inteins (Mills et al., 2014). Finally, steps 3 and 4 occur as in class 1 and 2 inteins.

### 2.3. Off-pathway cleavage reactions

Unproductive off-pathway reactions are also possible. The N-extein can be released from the intein prior to ligation to the C-extein in a process known as N-terminal cleavage. This type of cleavage is most often observed in inteins where the extein ligation reaction has been suppressed either by deliberate mutation of the +1 extein residue, or in inadvertent cases where the intein is splicing from a non-native context. Certain residues at the −1 position, in particular aspartate, as well as low pH, promote N-terminal cleavage (Amitai et al., 2009). Isolated C-terminal cleavage is mediated by succinimide formation at the C-terminus of the intein in the absence of extein ligation. As with N-terminal cleavage, the prevalence of C-terminal cleavage is strongly affected by the +1 extein residue, and also shows sensitivity to pH and temperature (Chong et al., 1996; Mathys et al., 1999; Wood et al., 1999). Although isolated cleavage reactions are commonly observed when inteins are moved to new contexts, and are deliberately stimulated in some intein applications, it is unknown to what degree off-pathway reactions occur in nature and if they are of any physiological consequences to intein-containing organisms.

## 3. Significance of inteins as environmental sensors

Inteins, many of which are also mobile genetic elements, are prevalent in microbes. They are present in about half of archaea and a quarter of bacteria, and more rarely in single-celled eukaryotes (Novikova et al., 2016). They are distributed sporadically and idiosyncratically, in ways that raised suspicion that they may be useful to their host cell, rather than simply selfish mobile genetic elements. Their occurrence is strongly favored in particular proteins, like those engaged in replication, recombination and repair, even in the non-orthologous replication functions of archaea and bacteria. Inteins also tend to cluster in specific regions of proteins, with a remarkable 70% occurring in ATP-binding domains of ATPases. This distinctive distribution suggests that inteins may provide benefit to their hosts, as posttranslational regulators. Inteins are indeed ideally equipped as sensors of their environment that then respond appropriately to either activate or inhibit host protein function (Belfort, 2017; Lennon and Belfort, 2017). This section reviews some of the conditions known to regulate protein splicing (Figure 1D), but is not exhaustive as additional environmental factors are known to control splicing.

### 3.1. Inteins as temperature sensors

When inteins act as sensors, they respond to environmental cues of various kinds, like temperature, salt concentration, pH, divalent metal ions and redox, to splice in response to these signals in a process termed conditional protein splicing (CPS) (Belfort, 2017; Lennon and Belfort, 2017). Their sensing function often relates to the environmental niche of the host organism. For example, CPS of inteins occurs in hyperthermophilic and halophilic archaea, where splicing is activated in response to heat and salt (Reitter et al., 2016), respectively. An example of an intein responding to the extracellular environment can be observed in the RadA recombinase from the archaeon *Pyrococcus horikoshii* (Topilina et al., 2015). Interactions between the intein and C-extein block splicing at temperatures <65°C, when the organism is under cold stress, and are ruptured as temperatures approach 100°C, the optimum growth temperature of the organism, when recombinase function would be required (Topilina et al., 2015). Intriguingly, splicing of RadA from the related archaeon *Thermococcus sibiericus* is activated by slightly lower temperatures, corresponding well to the optimum growth conditions for that organism (Lennon et al., 2018). Thus, the intein-as-sensor provides the means of regulating protein function under precise conditions that are beneficial to the organism, while also sparing ATP which is required to maximize growth rate.

### 3.2. Inteins as oxidative stress sensors

Alternatively, splicing may be blocked, to provide a protective role to the protein in which it resides. An example here is the oxidoreductase MoaA in the archaeon *Pyrococcus abyssi*, where the intein can form a covalent disulfide bond with the N-extein (Callahan et al., 2011). Under oxidizing conditions, this bond, formed between a cysteine in the N-extein and the catalytic Cys1 of the intein, blocks the first step of splicing. This appears to be more than mere coincidence, as oxygen is toxic to MoaA, leading to its inactivation. Under reducing conditions, the disulfide bond is broken, and splicing can proceed, such that active MoaA is produced. This is a beautiful example of disulfide trapping of precursor, that prevents active protein formation of this oxygen-labile enzyme, until a reducing environment becomes both conducive to and demanding of its activity. Then splicing is activated, when the threat to enzyme integrity has passed, thereby providing MoaA oxidoreductase activity when needed.

### 3.3. Inteins as DNA damage sensors

Another spectacular example of inteins sensing the intracellular environment is provided by the very *P. horikoshii* RadA recombinase mentioned above regarding the temperature dependence of splicing. Strikingly, single-stranded DNA *in vitro* or DNA damage *in vivo* promote faster and more accurate splicing of the RadA intein at all temperatures tested (Lennon et al., 2016). Those abovementioned interactions between the extein of the non-spliced precursor are disrupted by single-stranded DNA, thereby freeing the intein to splice. This finding is fascinating, given that single-stranded DNA is the substrate of RadA-recombinase, the ortholog of bacterial RecA and eukaryotic Rad51. This is the first example of substrate-activated splicing, to render the host protein active precisely at the time that it is required by the cell to perform homologous recombination or recombination-dependent replication.

### 3.4. Inteins as metal ion sensors

Single-celled eukaryotes also harbor inteins, in their chromosomal genes. The Prp8 protein, involved in RNA splicing, is a common site for intein insertion in fungi, as for example in the fungal pathogen *Cryptococcus neoformans*. Here, the divalent metal ions zinc and copper, which are important in the pathogenesis cycle, inhibit intein splicing (Green et al., 2019). This is achieved by these metals binding to active site residues in the intein. It has been known for many years that metal ions inhibit bacterial inteins (Mills and Paulus, 2001), as is the case with zinc, which is also important during mycobacterial pathogenesis. Mycobacteria can harbor inteins in up to four proteins, each of which can act as a sensor and switches to potentially regulate their lifestyles. The intein in DnaB replicative helicase of the model organism *Mycobacterium smegmatis* was shown to be zinc-responsive *in vivo* (Woods et al., 2020). This observation of control of DnaB function, and therefore presumably DNA replication, in the native host is significant, given that the previous studies of stress-induced intein regulation were all conducted *in vitro* or in non-native hosts.

## 4. Intein applications in basic research and biotechnology

The ability of inteins to rearrange peptide bonds within and between protein segments immediately suggested several applications in pure research and biotechnology, many of which have been reviewed extensively (Topilina and Mills, 2014; Sarmiento and Camarero, 2019). The ability to cleave and ligate peptides and protein segments allows engineering of new proteins and generate unique semisynthetic proteins. Controllable splicing and cleavage reactions are also used to activate proteins and enzymes under specific conditions *in vivo* and *in vitro*, allowing proteins to be used as cellular biosensors and in metabolic engineering (Di Ventura and Mootz, 2019). Split inteins are especially useful, and several orthogonal pairs have now been identified for applications in protein engineering and synthetic biology as well (Pinto et al., 2020). This section briefly covers several major applications of intein splicing and cleavage.

### 4.1. Self-removing affinity tags

One of the first recognized applications of inteins was in the development of self-removing affinity tags for recombinant protein purification, and this application has led to several issued patents (Chong et al., 1996; Mathys et al., 1999; Wood et al., 1999; Prabhala et al., 2022). In these methods, a modified intein is fused between a conventional affinity tag and desired target protein, where N-terminal or C-terminal intein junction residues are mutated to suppress the native splicing reaction. The residual off-pathway cleavage reactions are then exploited to selectively cleave the target protein from the tag-intein fusion, thereby providing an untagged target protein in a single affinity step. The first commercialized self-cleaving tag is the IMPACT-CN system from New England Biolabs, where the intein is induced to cleave at its N-terminus by thiol addition once the fusion had been purified. Alternate systems were also developed in which the C-terminal cleavage reaction is controlled by pH and/or temperature, which increased the number of applications for these tags, but with the unfortunate side effect of premature and unpredictable cleavage with different target proteins.

### 4.2. Assembly of semisynthetic proteins

The intein splicing reaction lends itself to several protein assembly methods, where two or more segments of a single mature peptide can be produced separately and assembled via protein trans-splicing (PTS) or expressed protein ligation (EPL). This capability allows short synthetic peptides, often with various labels or modifications, to be selectively incorporated into mature proteins. In EPL, the thioester formed during the first step of the canonical splicing mechanism is attacked by a nucleophilic cysteine residue at the N-terminus of a synthetic or intein derived peptide (Wang and Cole, 2020). In PTS, a split intein is used to splice two protein segments, which can be expressed in different host cells under different conditions. With both methods, mature proteins can be produced that contain different isotopic or chemical labels, as well as selectively localized posttranslational modifications. These approaches have greatly increased the size of proteins that can be examined by NMR and have provided new insights into how chemical modifications of proteins regulate their activity (Lim et al., 2020).

### 4.3. Molecular biosensors

In addition to the native biosensing capabilities listed above, both cis- and trans-splicing inteins have been fused to various recognition domains to act as biosensors. Several cis-splicing inteins have been modified by insertion of small molecule binding domains, where the presence of the small molecule (usually an estrogen or thyroid hormone) activates protein splicing and activation of a reporter enzyme (Sarmiento and Camarero, 2019). Although these controllable inteins can also be used in synthetic biology, they are limited by leaky splicing in the absence of the actuator molecule, and incomplete splicing when activated. The use of split inteins for biosensing has been more fully developed, where fused recognition domains are typically used to drive split inteins together to activate splicing. These systems have been developed to detect protein–protein interactions, protein translocation to cellular organelles, DNA modifications (through incorporation of polydactyl zinc finger domains), and the presence of different small molecules (Topilina and Mills, 2014).

### 4.4. Transgene delivery and regulation

A rapidly growing application of split inteins is in human gene therapy and the regulation of proteins in plants. A limitation in the delivery of gene therapies via AAV (Adeno-associated Virus) vectors is the small cargo size that they can carry. An exciting application of split inteins is to split a given therapeutic cargo and deliver it in fusion to split intein segments. Once delivered and translated, trans-splicing reconstitutes the active enzyme for therapeutic effect, and this method has been recently demonstrated in animal models (Tornabene et al., 2019). Split inteins are also being developed to reconstitute the relatively large CRISPR/Cas9 enzyme as part of novel gene editing applications (Truong et al., 2015). Split inteins have also been used to assemble and reconstitute proteins and enzymes in plants, where the use of the split intein is designed to reduce spread of the transgene into other plants. In this case, each segment of an herbicide resistance protein is fused to a split intein, and the two segments are inserted into different plant chromosomes. Under these conditions, co-inheritance of both segments is significantly reduced relative to the single-gene approach (Wang et al., 2014).

### 4.5. Additional applications

New inteins and intein applications are being developed in several laboratories around the world, where their modular design and robust ability to assemble and activate proteins are providing new mechanisms for research and tool development. Among the most exciting are synthetic metabolic circuits based on differential intein splicing (Wang et al., 2022), and the development of designer inteins for expanded applications in synthetic biology and cellular engineering (Burton et al., 2020). Split inteins have also been used to generate highly stable cyclic proteins, which may ultimately become a new platform scaffold for orally available protein drugs (Tavassoli, 2017).

## 5. Discussion

Over the past 30 years, our understanding of protein splicing has evolved from a simple mechanistic understanding to a fuller appreciation of inteins as beneficial regulatory elements and important tools for research and biotechnology applications. Observations of inteins evolving sensor activity that can regulate their host proteins in a beneficial way, coupled to their striking clustering to replication, recombination and repair proteins and ATPase domains, collectively suggest that inteins are retained because they confer a selective advantage to their host organisms. An eagerly awaited finding for the field is therefore that intein-containing organisms have a competitive edge in their native environments over their intein-less counterparts. To address this, future work must examine the importance of these elements within native intein-containing hosts to determine the impact of protein splicing, and the conditional inhibition thereof, on microbial physiology. In biotechnology, inteins have enabled new approaches for simple protein purification, basic protein structural research, metabolic engineering, and synthetic biology. Given the unique capabilities of these elements, as well as the frequency at which new applications emerge, the potential for intein-based applications is immense. Even in the emerging fields of novel medicines and gene therapy, inteins provide new and evolving strategies. For example, while inteins are present within several pathogens including *Mycobacterium tuberculosis* and *Cryptococcus neoformans*, they are absent from the genomes of humans. Inteins therefore represent attractive antimicrobial targets, whereby the inhibition of splicing could compromise pathogen survival (Wall et al., 2021; Tharappel et al., 2022). Excitingly, recent work has demonstrated that small molecules can target protein splicing as an antimicrobial strategy (Chan et al., 2016; Li et al., 2019, 2021). Therefore, despite being discovered over 30 years ago, inteins are still a very active area of research, and likely have additional biological functions and applications that have not yet been discovered.

## Author contributions

DW: Writing – original draft, Writing – review & editing. MB: Writing – original draft, Writing – review & editing. CL: Writing – original draft, Writing – review & editing.
